# A Birth and a Death

**Published:** 1870-01

**Authors:** 


### A BIRTH AND A DEATH.

BY DR. NED.

Twenty three years, this day, Mrs Julia H——, was honored by the arrival of a daughter. In less than twenty minutes the new comer was called Julia, for it was determined to have the name perpetuated.

There was unbounded joy in the elegant mansion of Mr. H., who, by the way, was something of a poet, and upon this occasion wrote as follows:

"One night as Old St. Peter slept,
He left the door of heaven ajar,
When through a little angel crept,
And came down with a falling star."

"God grant but this, I'll ask no more,
That when she leaves this world of pain,
She'll wing her way to that bright shore,
And find the door of heaven again."

Well, she has gone; her youth and beauty faded away, on her soft white cheek lingered the crimson hue, but alas! it was like the unnatural tint "that autumn paints upon the perished leaf." And Julia's history is the life picture of thousands. About ten minutes after she was named the old nurse, Aunt Betty, who had been in the business more than ten years and who perfectly understood all about babies and the the "rest of mankind"—as nurses generally do—threw the strings of her cap over her shoulders, and, with the border standing at an angle of at least, sixty degrees, seized the child and started for the usual ablution; and while it was kicking and screaming like fury, managed to hold its feet long enough to put a bandage, ten inches wide, upon its body, extending from the hips well up on the chest, and secured it there by five pins. She took great care that it was sufficiently snug to hold the ribs firmly and to their place, just as though there was danger of their getting away. Nature provided an elastic skin for this little body and put it on so loose as to allow the free motion of the lungs, but Aunt Betty knew better than that (queer old "dame") for she had been at it longer, and resolved that she would have the child done up snugly, or rather that she would finish up the job so "loosely" begun, in style. Next in order came the linen, worked and embroidered; then three skirts with ten inch bands, and each at least two yards long; and all secured with exactly five pins. At last the dress was produced

(if that is the proper name for it), but I should say it was a pair af arm holes, having about three yards of goods attached to them, and all these were firmly fixed upon the helpless little victim, who was now obliged to swallow a dose of castor oil—after which Aunt Betty declared "it was dressed and fixed up," ready to be introduced to the family and friends. Of course it could hardly breathe, and to kick was out of the question. In half an hour it had experienced a change of climate of at least forty degrees, and its arms was as blue as a whetstone; and when the oil began to gripe, little Julia began to scream. Aunt Betty diagnosed the case colic, and Mr. H. was started at once for a bottle of "Mrs Winslow's Soothing Syrup." He returned, almost out of breath, and down in the little stomach went pell mell, cracker, water, milk, sweet oil, catnep-tea, Mrs Winslow & Co. At last the baby had to yield, its delicate nature gave up the fight, and Aunt Betty took a hot sling. So between the sling and the anodyne, the nurse and baby slept till broad day light. Thus ended the operation of one evening to be repeated day after day, and in spite of all this kind of treatment the baby lived and grew. I have sat by the hour and watched Aunt Betty feed and stew. She would fix all sorts of things in milk—would put milk in every thing—and cram that poor child as long as it would hold a drop, until you would say the band must break. More than a hundred times I have seen the soup, porridge, or milk run out of her little mouth and Betty would always be ready to catch it with the spoon and tuck it right down her throat again. As soon as it was crammed down she began to trot the poor thing on her knee, a two-forty-gait, until up it would come, sour, curd; then, magnesia for acid stomach must be applied; and Mrs. Winslow for pain, while the bandage must be drawn up for fear the baby will do itself harm by crying. I am sure that by the time Julia was a year old, she had swallowed two dozen bottles of magnesia, as many more of syrup, and had had at least four hundred quarts of milk churned in her compressed stomach, by Aunt Betty's ceaseless trot, trot. Looking the matter over, I must say that beyond doubt, babies are the toughest of all created things. What they are put through and what is put through them, month after month, would kill a hearty, robust man in three weeks.

As soon as the child could sit alone, a high chair made its way into the house, and baby took her place at the table, began to eat what other folks did and before she was a year old, she would spend an extra half hour to finish her pork or ham. (a delicacy, by the way, unfit for any body to eat, for we read that the "devil entered" these creatures, and I think he went to *stay*, "milk for babies and meat for strong men;" but that is all well enough for old fashioned stomachs, or for the kind they had before the art of stomach-making was perfected.) Well, after the conflict of cutting teeth was over, and Julia had taken all sorts of compounds and mixtures, for worms and various other disorders, arising from a violated and outraged nature; as might be expected, she was a delicate creature. Her head, which had not been bandaged, was large enough for her age; but the chest and abdomen had been compressed from her first moment, and she looked as gaunt as a grey hound, and no more like a human being *should* look, than if she belonged to the ape family. *We* think it is barborous and cruel for the nobility of China to bind the feet of their children, and at the same time we allow our girls to have feet large enough to carry an entire family of "John Chinaman," and while they enjoy the luxury of feet, we destroy their bodies, and only ask transportation for a head and an enormous trail. In fact, we only permit freedom of gowth to the head, feet, frock, and waterfall. Julia was sent to a fashionable school and was asked to do as much as though she had enjoyed the luxury of a body. She had been to the dressmakers to get an outfit; and that artist discovered that nature had made several awful blunders, a few of which we will name.—In the first place, she put the skin too loose on the body; that was easily fixed with a cord and a corset. Then, she made the spinal column very flexible; that was remedied by whalebone. She had made the waist about six inches too short; that was arranged without trouble, for the bowels were fortunately not encased in bone, and they would yield to pressure—then again, she put on shoulders to hold up the dress and skirts; this was only a mistake, for she meant the hips all the time—a good joke. Lastly, she located the shoulder joint in the most unhandy and rediculous place, entirely too high, so it was removed to just above the elbow, in the exact point of use and beauty. In this infernal harness the poor girl worked until she graduated, at the age of twenty-one, and was then put under treatment for *consumption*. Yesterday she passed to the silent land of the sleepers. The pious and aged mother placed a beautiful wreath of flowers on her bosom, and the good pastor, who came to comfort the venerable parents, advised them that all this was but a *kind* Providence operating in a mysterous way for good; and they must not murmer, for "the Lord giveth and the Lord taketh away, &c." If it is bliss to be ignorant, I don't know that wisdom is of much use; but the facts are, that Julia was a victim, and died of bandages, medicine and fashion. No one should be charged with it but Aunt Betty, the medicine senders, and the dressmaker. It is unfair to lay all our errors and faults to Providence. It was suggested long ago to trust "Providence but to keep the powder dry," and I say if you want to enjoy the life God in his mercy has given you, you must not destroy the dwelling, force out the tenant, and then lay it to a beneficient Lord "who doeth all things well." Yours,

